# Supplementary material for: A clustering approach based on neuropsychological testing to detect early signs of probable MCI among community-dwelling older adults
Source: Front Aging Neurosci. 2026 Oct 12;18:1926431. doi: 10.3389/fnagi.2026.1926431 (PMC13696466; doi:10.3389/fnagi.2026.1926431)
Supplement: Supplementary file 1 [file supplementary_file_1.docx]

**S1 – Supplementary Figure 1 Dendrogram of Hierarchical Clustering based on the Ward’s criterion.**

The height of the branches indicates the dissimilarity between clusters. The dendrogram was partitioned (black horizontal line) to maximize the distance between nodes. Cluster solution is indicated by three colored groups.
