# Supplementary material for: A clustering approach based on neuropsychological testing to detect early signs of probable MCI among community-dwelling older adults
Source: Front Aging Neurosci. 2026 Oct 12;18:1926431. doi: 10.3389/fnagi.2026.1926431 (PMC13696466; doi:10.3389/fnagi.2026.1926431)
Supplement: Supplementary file 2 [file supplementary_file_2.docx]

**Supplementary results**

To provide external clinical validation without circularity, a supplementary analysis was performed on a subsample of the first 60 participants included (general characteristics are visible in Table 1 page 2) using the Walking Trail Making Test (WTMT). The WTMT is the cognitive-locomotor adaptation of TMT proposed by Alexander et al., (2005), containing 3 conditions of increasing complexity: (i) W-TMT-N, with only numbers from 1 to 20 and where participants have to walk on the 20 numeric targets in increasing order; (ii) W-TMT-A, with the same 20 numbers as in W-TMT-N but with 20 other numeric distractors that the participants have to avoid while walking; (iii) W-TMT-B, with both numbers and letters as targets, where participants have to walk on the targets alternating between numbers and letters in increasing order for numbers and alphabetical order for letters (i.e., 1–A–2–B–3–C, etc.) while avoiding 20 other numeric and alphabetic distractors. In the MCI population with a confirmed diagnosis, the WTMT-B has been shown to accurately identify dysexecutive MCI with 100% sensitivity and 80% specificity (Perrochon and Kemoun, 2014).

An a priori power calculation based on the large effect sizes (*d*=1.11) reported in the study of (Persad et al., 2008) confirmed that a minimum of 54 participants (18 per group) is required to achieve adequate statistical power (90%) across three groups. Thus, our subsample (n=60) provided robust statistical power for this external validation. Following previous recommendations (Alexander et al., 2005; Persad et al., 2008; Perrochon and Kemoun, 2014), WTMT-B execution times were compared across clusters using an ANCOVA corrected by spontaneous walking speed to isolate the cognitive-motor interference cost from basic locomotor ability.

A significant cluster effect was found (*F*_2,56_=7.32, *p*=.001, *η^2^_p_*=.21, large effect). The *post-hoc* comparisons showed a significant difference between clusters 1 and 2 (respectively, M=18.40, SD=6.72 s *vs* M=28.41, SD=14.08 s; *t*_56_=-3.33, *p*=.004, *d*=-0.99, large effect) and between clusters 1 and 3 (respectively, M=18.40, SD=6.72 s *vs* M=28.97, SD=8.05 s; *t*_56_=-2.79, *p*=.019, *d*=-1.06, large effect) (see Figure 1 page 2).

**Table 1. Characteristics of the sub-sample used in the external validation analysis for each cluster.**

| Variable | Cluster sub-sample (N=60) | | |
| --- | --- | --- | --- |
|  | Cluster 1 (n=32) | Cluster 2 (n=19) | Cluster 3 (n=9) |
| Age (years) | 72.15±4.17  [65.00; 81.08] | 80.64±4.12  [72.67; 88.83] | 81.16±2.67  [78.08; 86.92] |
| MoCA (score /30) | 26.97±1.93  [21; 30] | 24.42±2.41  [18; 29] | 20.22±2.68  [18; 24] |
| MIS (score /15) | 12.16±3.17  [3; 15] | 9.53±2.82  [3; 14] | 3.44±2.92  [0; 7] |
| Delta-TMT (s) | 30.96±19.92  [5.70; 77.50] | 62.28±33.59  [5.00; 130.60] | 86.49±45.38  [42.40; 192.50] |
| Inter-Hi | 2.02±0.51  [1.20; 3.13] | 2.55±0.86  [1.48; 5.28] | 2.62±1.07  [1.42; 4.95] |

Values are mean ± standard deviation [Minimum; Maximum]

MOCA = Montreal Cognitive Assessment.

MIS= Memory index score.

Delta-TMT =Trail making test (Part B - Part A).

Inter-Hi=The high interference ratio (Stroop-I/Stroop-C).

**Figure 1. Boxplots representing post-hoc comparisons between clusters from hierarchical clustering on principal component analysis in a sub-sample of 60 participants on WTMT-B performance.**

**References.**

Alexander, N. B., Ashton-Miller, J. A., Giordani, B., Guire, K., and Schultz, A. B. (2005). Age differences in timed accurate stepping with increasing cognitive and visual demand: A walking trail making test. *J. Gerontol. A. Biol. Sci. Med. Sci.* 60, 1558–1562. doi: 10.1093/gerona/60.12.1558

Perrochon, A., and Kemoun, G. (2014). The walking trail-making test is an early detection tool for mild cognitive impairment. *Clin. Interv. Aging* 9, 111–119. doi: 10.2147/CIA.S53645

Persad, C. C., Jones, J. L., Ashton-miller, J. a, Alexander, N. B., and Giordani, B. (2008). Executive function and gait in older adults with cognitive impairment. *J. Gerontol. A. Biol. Sci. Med. Sci.* 63, 1350–1355. doi: 10.1093/gerona/63.12.1350
