# Supplementary material for: A clustering approach based on neuropsychological testing to detect early signs of probable MCI among community-dwelling older adults
Source: Front Aging Neurosci. 2026 Oct 12;18:1926431. doi: 10.3389/fnagi.2026.1926431 (PMC13696466; doi:10.3389/fnagi.2026.1926431)
Supplement: Supplementary file 3 [file table_1.docx]

**S2 – Supplementary Table 1: Distribution of normative classes for the three cluster in each condition of all neuropsychological tests.**

| Test | Condition | Category | Cluster 1  (n=82) |  |  | Cluster 2  (n=56) |  |  | Cluster 3  (n=23) |  | *χ*^2^_8_ (*p*) |
| --- | --- | --- | --- | --- | --- | --- | --- | --- | --- | --- | --- |
|  |  |  | n | % |  | n | % |  | n | % |  |
| MoCA | MoCA Score | Deficit | 3 | 3.66 |  | 10 | 17.86 |  | 9 | 39.13 | 40.20 (<.001) |
|  |  | Inferior | 9 | 10.98 |  | 13 | 23.21 |  | 8 | 34.78 |  |
|  |  | Average | 46 | 56.10 |  | 28 | 50.00 |  | 6 | 26.09 |  |
|  |  | Superior | 15 | 18.29 |  | 4 | 7.14 |  | 0 | 0.00 |  |
|  |  | Very Superior | 9 | 10.98 |  | 1 | 1.79 |  | 0 | 0.00 |  |
|  | MIS | Deficit | 0 | 0.00 |  | 0 | 0.00 |  | 4 | 17.39 | 54.73 (<.001) |
|  |  | Inferior | 3 | 3.66 |  | 1 | 1.79 |  | 5 | 21.74 |  |
|  |  | Average | 32 | 39.02 |  | 31 | 55.36 |  | 14 | 60.87 |  |
|  |  | Superior | 45 | 54.88 |  | 21 | 37.50 |  | 0 | 0.00 |  |
|  |  | Very Superior | 2 | 2.44 |  | 3 | 5.36 |  | 0 | 0.00 |  |
| TMT | TMT-A | Deficit | 0 | 0.00 |  | 0 | 0.00 |  | 0 | 0.00 | *17.40 (<.005) |
|  |  | Inferior | 3 | 3.66 |  | 5 | 8.93 |  | 1 | 4.35 |  |
|  |  | Average | 10 | 12.20 |  | 9 | 16.07 |  | 11 | 47.83 |  |
|  |  | Superior | 69 | 84.15 |  | 42 | 75.00 |  | 11 | 47.83 |  |
|  | TMT-B | Deficit | 0 | 0.00 |  | 0 | 0.00 |  | 2 | 8.70 | ^$^43.88 (<.001) |
|  |  | Inferior | 3 | 3.66 |  | 4 | 7.14 |  | 5 | 21.74 |  |
|  |  | Average | 20 | 24.39 |  | 30 | 53.57 |  | 13 | 56.52 |  |
|  |  | Superior | 59 | 71.95 |  | 22 | 39.29 |  | 3 | 13.04 |  |
|  | Delta-TMT | Deficit | 0 | 0.00 |  | 2 | 3.57 |  | 4 | 17.39 | ^$^42.84 (<.001) |
|  |  | Inferior | 9 | 10.98 |  | 8 | 14.29 |  | 8 | 34.78 |  |
|  |  | Average | 19 | 23.17 |  | 28 | 50.00 |  | 8 | 34.78 |  |
|  |  | Superior | 54 | 65.85 |  | 18 | 32.14 |  | 3 | 13.04 |  |
| V-ST | Stroop-C | Deficit | 4 | 4.88 |  | 2 | 3.57 |  | 1 | 4.35 | 13.32 (.101) |
|  |  | Inferior | 7 | 8.54 |  | 3 | 5.36 |  | 3 | 13.04 |  |
|  |  | Average | 48 | 58.54 |  | 24 | 42.86 |  | 15 | 65.22 |  |
|  |  | Superior | 21 | 25.61 |  | 19 | 33.93 |  | 3 | 13.04 |  |
|  |  | Very Superior | 2 | 2.44 |  | 8 | 14.29 |  | 1 | 4.35 |  |
|  | Stroop-W | Deficit | 5 | 6.10 |  | 2 | 3.57 |  | 2 | 8.70 | 8.15 (.419) |
|  |  | Inferior | 9 | 10.98 |  | 6 | 10.71 |  | 5 | 21.74 |  |
|  |  | Average | 56 | 68.29 |  | 31 | 55.36 |  | 12 | 52.17 |  |
|  |  | Superior | 9 | 10.98 |  | 13 | 23.21 |  | 3 | 13.04 |  |
|  |  | Very Superior | 3 | 3.66 |  | 4 | 7.14 |  | 1 | 4.35 |  |
|  | Stroop-I | Deficit | 0 | 0.00 |  | 1 | 1.79 |  | 1 | 4.35 | 13.31 (.093) |
|  |  | Inferior | 1 | 1.22 |  | 6 | 10.71 |  | 2 | 8.70 |  |
|  |  | Average | 53 | 64.63 |  | 26 | 46.43 |  | 13 | 56.52 |  |
|  |  | Superior | 17 | 20.73 |  | 18 | 32.14 |  | 4 | 17.39 |  |
|  |  | Very Superior | 11 | 13.41 |  | 5 | 8.93 |  | 3 | 13.04 |  |
|  | Inter-Low | Deficit | 1 | 1.22 |  | 3 | 5.36 |  | 1 | 4.35 | 10.92 (.206) |
|  |  | Inferior | 3 | 3.66 |  | 8 | 14.29 |  | 2 | 8.70 |  |
|  |  | Average | 56 | 68.29 |  | 33 | 58.93 |  | 12 | 52.17 |  |
|  |  | Superior | 16 | 19.51 |  | 7 | 12.50 |  | 7 | 30.43 |  |
|  |  | Very Superior | 6 | 7.32 |  | 5 | 8.93 |  | 1 | 4.35 |  |
|  | Inter-Hi | Deficit | 0 | 0.00 |  | 3 | 5.36 |  | 1 | 4.35 | 14.31 (.807) |
|  |  | Inferior | 5 | 6.10 |  | 8 | 14.29 |  | 2 | 8.70 |  |
|  |  | Average | 45 | 54.88 |  | 32 | 57.14 |  | 15 | 65.22 |  |
|  |  | Superior | 24 | 29.27 |  | 12 | 21.43 |  | 2 | 8.70 |  |
|  |  | Very Superior | 8 | 9.76 |  | 1 | 1.79 |  | 3 | 13.04 |  |

*Note*. “Deficit” class was defined for MoCA, MIS and V-ST by a Z-score inferior or equal to -1.65 and for the TMT by a performance below or equal to the 5^th^ percentile. *Degrees of freedom for TMT-A are 4; ^$^Degrees of freedom for TMT-B and Delta TMT are 6.
